# Synergistic Effect of 3D/2D Vanadium Diselenide/Tungsten Diselenide Hybrid Materials: Electrochemical Detection of 5-Nitroquinoline a Hazard to the Aquatic Environment

**DOI:** 10.1021/acsami.4c02412

**Published:** 2024-06-17

**Authors:** Ramaraj Sukanya, Prajakta R. Chavan, Raj Karthik, Mahmudul Hasan, Jae-Jin Shim, Carmel B. Breslin

**Affiliations:** † Department of Chemistry, Maynooth University, Maynooth, Co. Kildare W23F2H6, Ireland; ‡ School of Chemical Engineering, Yeungnam University, Gyeongsan, Gyeongbuk 38541, The Republic of Korea; § Centre of Molecular Medicine and Diagnostics (COMManD), Department of Biochemistry, Saveetha Dental College and Hospitals, Saveetha Institute of Medical and Technical Sciences (SIMATS), Saveetha University, Chennai 600 077, India

**Keywords:** multidimensional, 2D-nanosheets, 3D-microspheres, toxic to the aquatic environment, 5-nitroquinoline

## Abstract

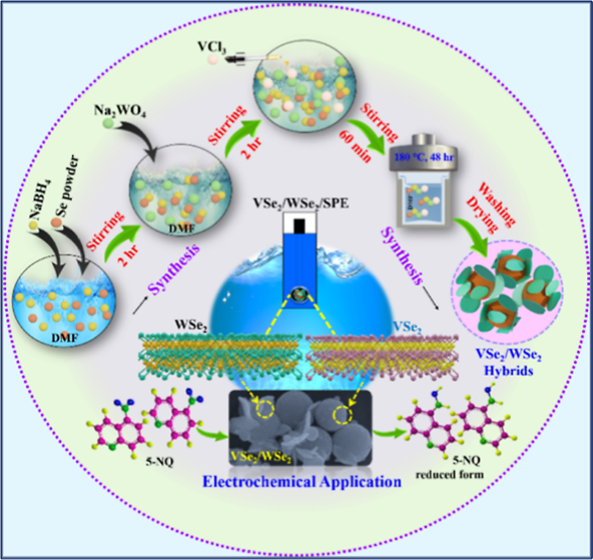

The development of multidimensional structured electrode materials with simple synthetic methods and their electrochemical sensing ability against environmental pollution is still a challenge. In this article, we propose a hybrid formed using multidimensional (3D/2D) vanadium diselenide microspheres and tungsten diselenide nanosheets (VSe_2_/WSe_2_) for the electrochemical detection of 5-nitroquinoline (5-NQ), a highly toxic and hazardous substance that is polluting aquatic life due to increasing industrial activities. The 3D/2D VSe_2_/WSe_2_ hybrids were prepared by a simple solvothermal method and their morphological and structural analysis was confirmed by various spectroscopy and analytical techniques such as powder X-ray diffraction, X-ray photoelectron spectroscopy, field emission scanning electron microscopy–energy dispersive X-ray spectroscopy, transmission electron microscopy, cyclic voltammetry, and differential pulse voltammetry. The proposed 3D/2D architecture showed a strong synergistic effect between the two components as well as high electrical conductivity. As a result, an increased peak current for the reduction and detection of 5-NQ was achieved compared to other modified and unmodified disposable screen-printed electrodes (SPE), such as bare SPE, VSe_2_/SPE, and WSe_2_/SPE. Under the optimized electrochemical conditions, VSe_2_/WSe_2_/SPE showed large linear response ranges (0.012–1053, 1183–3474 μM), a low detection limit (0.002 μM), good sensitivity along with good selectivity, and repeatability for the detection of 5-NQ. With this prominent electrochemical behavior, the VSe_2_/WSe_2_ electrode has clear potential to produce high-performance sensor devices.

## 1 Introduction

Nowadays, transition-metal chalcogenides (TMDs) are considered efficient materials for various electrochemical applications.^1^ In particular, the two-dimensional transition metal selenides (2D-TMSs) have been described as highly active electrode materials for electrochemical sensing platforms due to their high electrocatalytic activity and chemical stability.^2^ The presence of unsaturated electroactive edge sites, the high metallic bonding with transition metals, and the low electronegativity of selenium (Se) in TMS play an important role in enhancing the electrochemical activity of the sensing process.^3^ As typical TMS types, NiSe_2_, MnSe_2_, WSe_2_, and VSe_2_ have already established themselves as well-known candidates for electrochemical sensors due to their low cost and intrinsic activity.^4−7^ Besides their potential as electrochemical sensors, VSe_2_ and WSe_2_ have been recently developed for other applications, mainly due to their large interlayer spacing and good redox properties.^8,9^ VSe_2_ is an important TMD that is characterized by its sandwich-like layered structure. Compared to other TMDs, VSe_2_ has gained more attention due to its high metallic behavior in both polymorphisms such as the 1T and 2H phases. This higher metallic property of VSe_2_ is due to the electronic coupling interaction of V^4+^-V^4+^ pairs, which can lead to higher conductivity and improved electrochemical activity.^10,11^ In addition, the large interlayer spacing of VSe_2_ provides more active catalytic sites and short ion transport channels to improve the redox process.^8^ Furthermore, the presence of multiple oxidation states of V promotes the redox process and generates strong changes in the electronic structure, which can lead to a strong electronic interaction between V and Se, thereby improving the electrochemical performance.^8^ Although VSe_2_ has been used as an electrode material for the electrochemical sensing of analytes,^7^ the electrochemical properties of VSe_2_ have not been extensively explored, and its use is limited by the difficulties in synthesizing various nanostructures of VSe_2_. In addition, V^4+^ is unstable and can oxidize or reduce to other oxidation states (either V^3+^ or V^5+^), which can affect the electrochemical performance and stability of the device.^11^ To overcome these problems, several dimensional or multiple hybrid structures such as 1D/2D or 2D/3D have been developed and used for various electrochemical studies.^12^ Moreover, the development of multidimensional hybrids leads to stronger synergistic effects than single materials. Remarkably, the design of 2D/3D architectures leads to high structural stability and improved charge transport properties.

Like VSe_2_, WSe_2_ also belongs to the family of TMDs and is used as an electrode material for various electrochemical studies due to its layered structure.^13^ This layered structure is in the form of Se–W–Se and consists of a W atomic layer surrounded by two Se atomic layers. In addition, the layered structure offers several advantages such as a large surface area, high chemical and mechanical strength, and tunable electronic properties.^14^ In addition, the layered WSe_2_ provides more active catalytic sites and enables fast charge transport, which improves the electrochemical response. At the same time, the strong interaction between the WSe_2_ layers causes them to easily overlap and limit the number of active sites, which can lead to a decrease in the electrochemical performance.^15^ To solve this problem, various strategies have been developed to improve the electrochemical performance of WSe_2_, which are categorized into different types: (i) doping with metals (transition or rare-earth metals), (ii) synergy with carbon materials, and (iii) construction of hybrids with other metal composites (1D/2D or 2D/3D combinations).^15−17^ In the construction of hybrids with other metal selenides, the electrochemical properties can be improved by introducing different structures. In particular, the combination of 3D and 2D multiple structures leads to strong synergistic effects, as the different properties, in terms of the stability of 3D and the fast charge transport of the layered 2D structures, can lead to improved electrochemical performance.^12^ Encouraged by these combinations, we propose the construction of a 3D/2D VSe_2_/WSe_2_ hybrid structure as a selective electrode material for the electrochemical detection of environmental pollutants.

The environmental pollutant chosen was 5-nitroquinoline (5-NQ), which belongs to the family of polyaromatic heterocyclic compounds. It is known that quinoline-based water pollutants are formed during the decomposition of incomplete combustion processes and pyrolysis of biomass products (gasoline and fuels).^18^ Due to its toxicity, 5-NQ is considered mutagenic or carcinogenic. In particular, the presence of a highly reducible nitro group in 5-NQ leads to a stronger carcinogenic and mutagenic effect on humans and environmental factors.^19^ Therefore, the analysis of 5-NQ in various environmental samples is of great importance for the evaluation of its toxicity and the control of environmental pollution. For this purpose, electrochemical sensing of 5-NQ has been applied, which is a more effective and sensitive technique than other methods.^20^ However, the choice of electrode material determines the ease of detection of 5-NQ in various pharmaceutical and environmental samples.^21^ In our previous report, we introduced CoSe/Ni_3_B/SPCE for the detection of 5-NQ and obtained a low detection limit and good recovery results in various environmental samples.^20^ Based on the above study, we believe that the VSe_2_/WSe_2_ hybrid also has the potential to function as an active electrode material for 5-NQ in various environmental samples.

In this work, we have attempted to fabricate a 3D/2D VSe_2_/WSe_2_ hybrid structure for the electrochemical sensing of 5-NQ. For this purpose, the VSe_2_/WSe_2_ hybrid was prepared by simple solvothermal synthesis. Subsequently, the prepared VSe_2_/WSe_2_ hybrid was characterized by various analytical and spectroscopic techniques, including p-XRD, field emission scanning electron microscopy (FE-SEM), transmission electron microscopy (TEM), and X-ray photoelectron spectroscopy (XPS) analyses. After confirmation of the hybrid structure, the VSe_2_/WSe_2_ was used as an electrocatalyst for the sensing of 5-NQ by applying it to a screen-printed electrode (SPE). The VSe_2_/WSe_2_/SPE was investigated for its electrochemical properties and sensing behavior using different voltammetric techniques. Subsequently, the VSe_2_/WSe_2_/SPE was used for the quantification of 5-NQ using differential pulse voltammetry (DPV) analysis, and the results showed that the VSe_2_/WSe_2_/SPE exhibited excellent electrochemical sensing behavior.

## 2 Experiment Section

### 2.1 Chemicals and Reagents

Se powder (Se), sodium borohydride (NaBH_4_), sodium tungstate dihydrate (Na_2_WO_4_·2H_2_O, 99%), vanadium chloride (VCl_3_, 97%), 5-nitroquinoline (5-NQ), and *N*,*N*-dimethylformamide (DMF) were purchased from Sigma-Aldrich (https://www.sigmaaldrich.com/KR/en), Alfa Aesar, and Samchun chemical companies, South Korea. The purchased chemicals were used directly for synthesis and electrochemical tests without further treatment and purification. The required 0.05 M phosphate buffer (0.05 M PB) electrolyte was prepared by mixing NaH_2_PO_4_ and Na_2_HPO_4_. Deionized water (DI), which is mandatory for electrochemical tests, was used for the preparation of all required test solutions.

### 2.2 Characterization Techniques

The morphology, phase orientation, and chemical states of the synthesized VSe_2_/WSe_2_ hybrids and WSe_2_ nanosheets were characterized by field emission scanning electron microscopy (FE-SEM, Hitachi S-4800, attached with energy dispersive X-ray spectroscopy (EDAX)), transmission electron microscopy (TEM, FEI Tecnai G^2^ F20, acceleration voltage-200 kV), powder X-ray diffraction (p-XRD, PANalytical, X’Pert-PRO MPD X-ray diffractometer under high-intensity Cu Kα radiation (λ:1.5406 Å, current: 30.0 mA and voltage: 40.0 kV)), and X-ray photoelectron spectroscopy (XPS, Thermo Scientific Instruments, using a monochromatic Al–Kα X-ray source).

The electrochemical analyses, such as cyclic voltammetry (CV) and differential pulse voltammetry (DPV) were performed with an Autolab potentiostat/galvanostat (Metrohm PGSTAT 302 N instrument, Netherlands), using a three-electrode cell system with screen-printed carbon (SPE, was purchased from Zensor R&D Co, Ltd., Taiwan (www.zensorrd.com/PrintedElectrodes.html)), platinum wire and saturated calomel electrode as working, counter, and reference electrodes, respectively.

### 2.3 Solvothermal Synthesis of 2D-WSe_2_ Nanosheets and VSe_2_/WSe_2_ (3D/2D) Hybrids

**Scheme 1:**
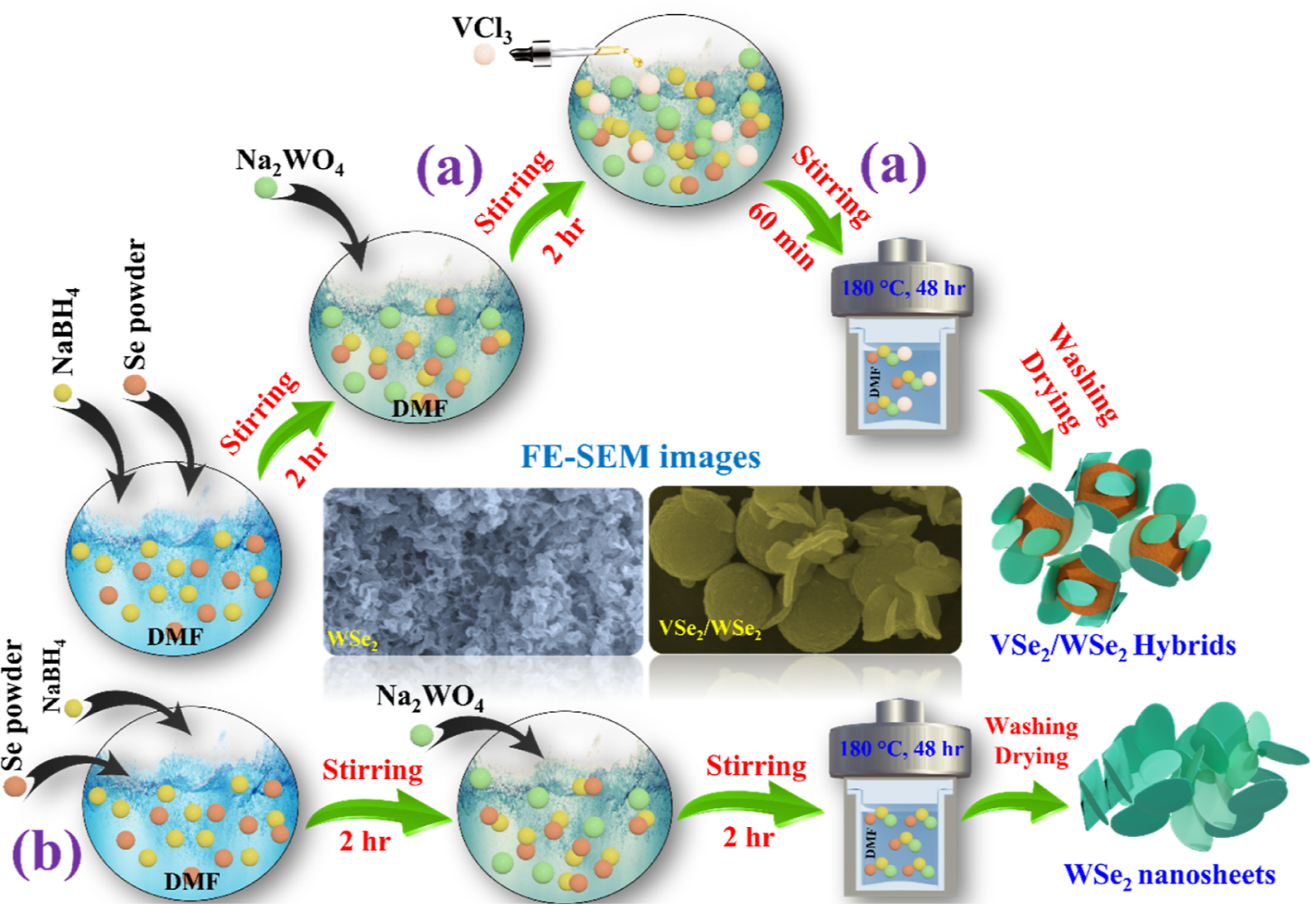
Schematic Representation of the Synthesis of WSe_2_ Nanosheets and VSe_2_/WSe_2_ Hybrids, where (a) Describes the Fabrication of the VSe_2_/WSe_2_ Hybrids and (b) Details the Formation of the WSe_2_ Nanosheets

2D-WSe_2_ nanosheets and VSe_2_/WSe_2_ hybrids were prepared using the solvothermal method. For the preparation of VSe_2_/WSe_2_ hybrids, 0.022 g NaBH_4,_ and 0.075 g Se powder were first dissolved together in 50 mL DMF with vigorous stirring for 2 h at room temperature. Then, 0.158 g NaWO_4_ was slowly added to the above mixture, and the solution was stirred again for another 2 h. Later, 0.075 g VCl_3_ was dissolved separately in 10 mL DMF and added dropwise to the mixture, which was then stirred for another hour. Finally, the solution (60 mL) was transferred to the reactor, where it was maintained at 180 °C for 48 h. Subsequently, it was cooled to room temperature, and the resulting product was precipitated by centrifugation at 10,000 rpm for 10 min. Later it was thoroughly washed with DI and ethanol and dried overnight at 50 °C in a vacuum oven. The overall synthetic procedure of the VSe_2_/WSe_2_ (3D/2D) hybrids is shown in Scheme 1a. For comparative studies, 2D-WSe_2_ nanosheets were synthesized using the same procedure as described in Scheme 1b, but without the addition of VCl_3_. Additionally, VSe_2_ and various ratios of V (1.5:1) in VSe_2_/WSe_2_ were prepared using the same procedure, both with and without the addition of sodium tungstate.

### 2.4 Preparation of VSe_2_/WSe_2_ Hybrids and Sensor Fabrication

For the formulation of the working electrode, the commercially available SPE was used. Before modification, the SPE surface was washed well with DI water and ethanol to remove any surface contaminants. The surface cleaned SPE was dried with N_2_ gas for later modification. To prepare a VSe_2_/WSe_2_ suspension, approximately 3 mg of VSe_2_/WSe_2_ (optimized) was dispersed in 0.5 mL DI water and sonicated for 15 min to obtain a uniform suspension. Finally, an aliquot of 8 μL (optimized) of the prepared suspension was placed as droplets on the SPE surface to prepare VSe_2_/WSe_2_/SPE, and then the water was evaporated in an air oven at ambient temperature. The dried VSe_2_/WSe_2_/SPE was used for the electrochemical detection of 5-NQ. The same procedure was used to prepare WSe_2_/SPE and VSe_2_/SPE as control electrodes for 5-NQ sensing.

## 3 Results and Discussion

### 3.1 Structural and Morphological Analysis of the Materials

**Figure 1:**
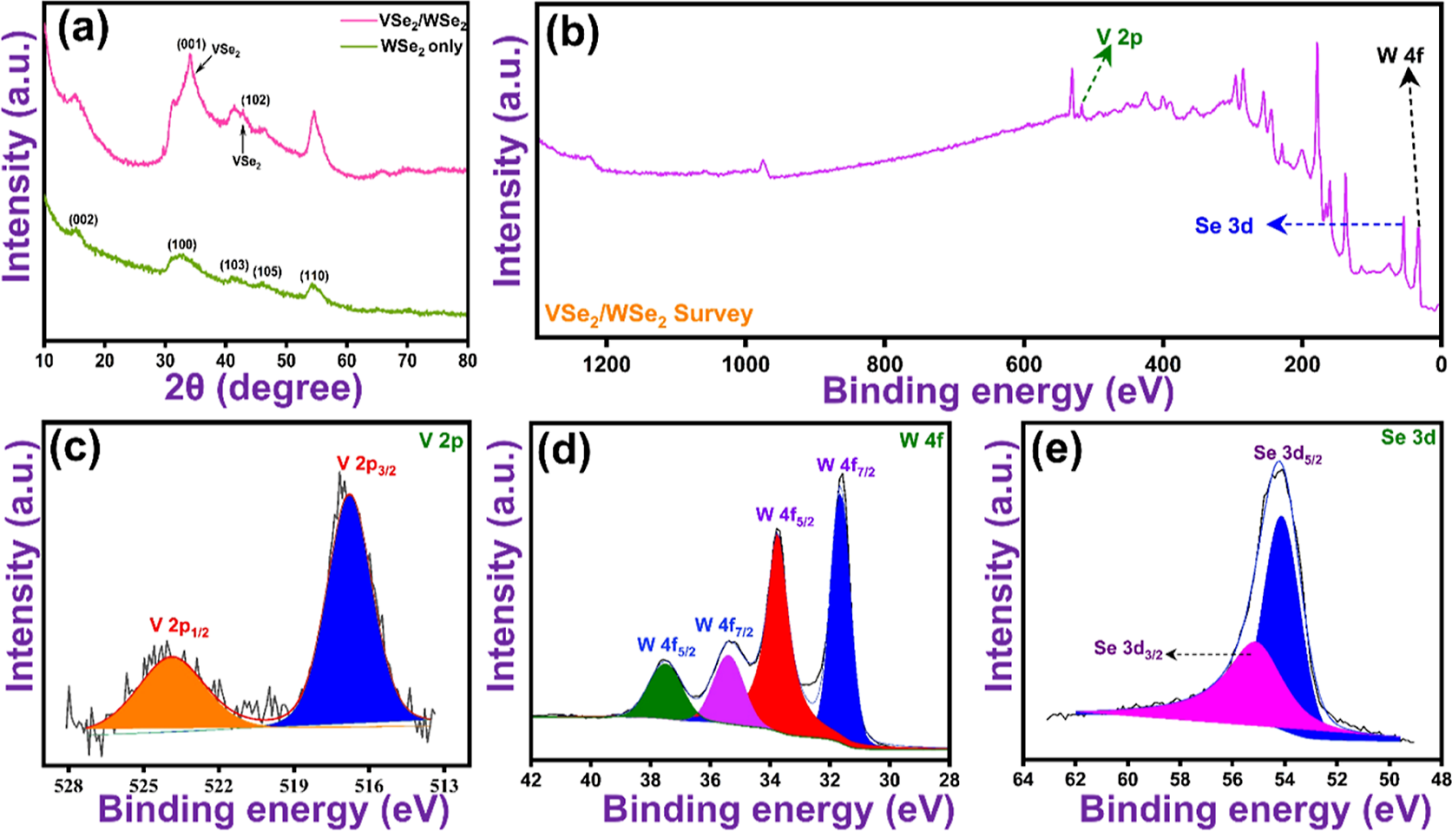
(a) XRD diffractogram of WSe_2_, and VSe_2_/WSe_2_. (b) Survey spectrum, and high-resolution spectra of (c) V 2p, (d) W 4f, and (e) Se 3d of VSe_2_/WSe_2_.

To confirm the formation of the 3D/2D VSe_2_/WSe_2_ hybrid, various spectroscopic studies were performed. First, the crystallographic feature of VSe_2_/WSe_2_ was confirmed by p-XRD analysis. The corresponding XRD patterns for the pristine WSe_2_ and VSe_2_/WSe_2_ samples are shown in Figure 1a. The reference powder XRD patterns for VSe_2_/WSe_2_ adapted from the ICSD database are shown in Figure S1(a). The XRD pattern of pristine WSe_2_ confirms the presence of multiple diffraction peaks at angles of 15.1, 32.7, 41.2, 45.8, and 54.2°, which correspond to the lattice plane reflections of (002), (100), (103), (105), and (110), respectively. These planes belong to the hexagonal WSe_2_ with a *P*6_3_/*mmc* space group and agree well with the standard reference pattern of JCPDS no. 38-1388.^22,23^ The presence of low diffraction reflections indicates the polycrystalline nature of the prepared hexagonal WSe_2_. In addition, the presence of a broad reflection in the (002) plane suggests that most of the crystallization occurred in the *z*-direction to form 2H–WSe_2_ layers. These weak and broad diffraction peaks indicate that the WSe_2_ layers were packed in an extremely disordered arrangement with low stacking.^22^ After combining with VSe_2_ to give VSe_2_/WSe_2_, changes in the XRD pattern were observed, as shown in Figure 1a. It can be seen from the figure that the obtained XRD pattern confirms the presence of a mixed phase of VSe_2_ and WSe_2_. In particular, the high-intensity diffraction angles with a value of 34.1 and 42.0° with respect to the lattice planes of (001) and (102) confirm the presence of VSe_2_. The above diffraction planes are in good agreement with the previously reported metallic VSe_2_ (JCPDS no. 89-1641).^24^ On the other hand, the main diffraction peaks of pristine WSe_2_ can also be seen in the XRD pattern of the hybrid in Figure 1a. A slight shift of all diffraction peak angles is observed, indicating a strong synergistic effect between VSe_2_ and WSe_2_ in the final hybrid structure. From the XRD analysis, it is evident that the VSe_2_/WSe_2_ has low crystallinity. Moreover, different molar ratios of V in VSe_2_/WSe_2_ were prepared, and the XRD results are provided in Figure S1b. From the figure, it is evident that the intensity of VSe_2_ increases with an increase in the molar ratio up to 1.5. Notably, there are no changes in the phase of WSe_2_, although its peak intensity is lower compared to the 1:1 ratio of the VSe_2_/WSe_2_ hybrids.

In addition, XPS surface analysis was performed to estimate the electronic states and binding energies of the VSe_2_/WSe_2_ hybrid. The corresponding XPS survey and individual scan spectra are shown in Figure 1b–e. The survey spectrum in Figure 1b confirms the presence of vanadium (V), tungsten (W), and selenium (Se) in the hybrid structure. The high-resolution spectrum of V in Figure 1c shows the presence of two deconvoluted peaks at 516.7 and 523.8 eV, corresponding to the V 2p_3/2_ and V 2p_1/2_ subshells, and confirms the presence of the V^4+^ state in the hybrid structure.^25^ The XPS spectrum of W in Figure 1d shows four deconvoluted peaks with binding energies of 31.6, 33.7, 35.3, and 37.5 eV, confirming the presence of mixed oxidation states of W^4+^ (4f_7/2_ and 4f_5/2_) and W^6+^ (4f_7/2_ and 4f_5/2_) in the final hybrid. Compared to previously reported studies,^26,27^ the above-mentioned binding energies of W show a slight shift in the peak values, indicating a significant transfer of electrons from W to V in the final hybrid system. This shift in peak position represents a strong electronic interaction between VSe_2_ and WSe_2_. The XPS spectrum in the selenium region shows doublet peaks at 54.1 and 55.1 eV, which were assigned to Se 3d_5/2_ and Se 3d_3/2_, respectively.^28^ Compared to previously reported metal selenides,^28^ the XPS spectrum of Se in VSe_2_/WSe_2_ also shows reduced peak splitting and a broader area, indicating the successful formation of a hybrid. Finally, the XPS results confirm the occurrence of phase and electronic changes after the formation of the VSe_2_/WSe_2_ hybrid.

**Figure 2:**
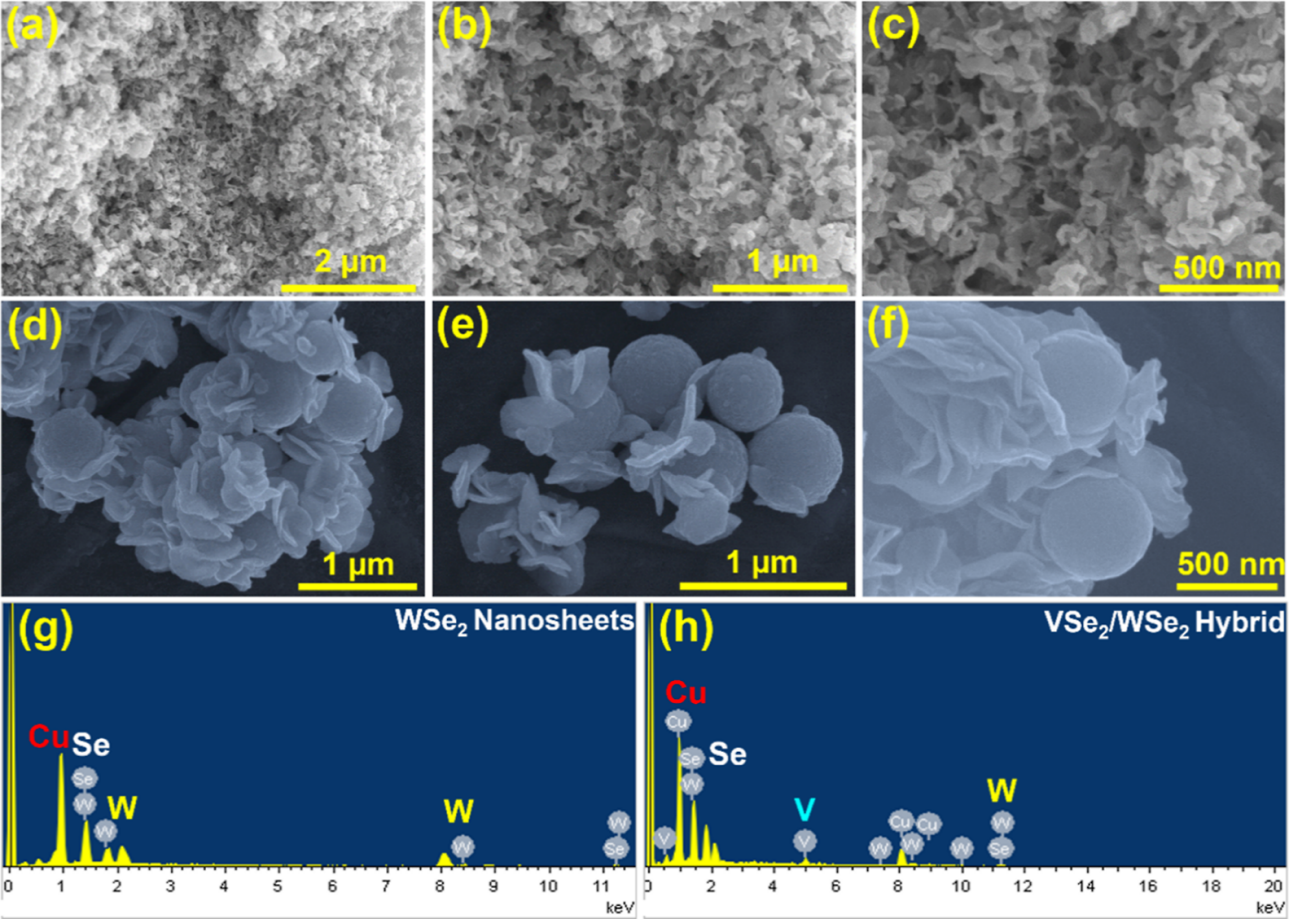
Different magnified FE-SEM images of (a–c) WSe_2_, and (d–f) VSe_2_/WSe_2_ hybrid. EDAX spectra of (g) WSe_2_ nanosheets, and (h) VSe_2_/WSe_2_ hybrid.

**Figure 3:**
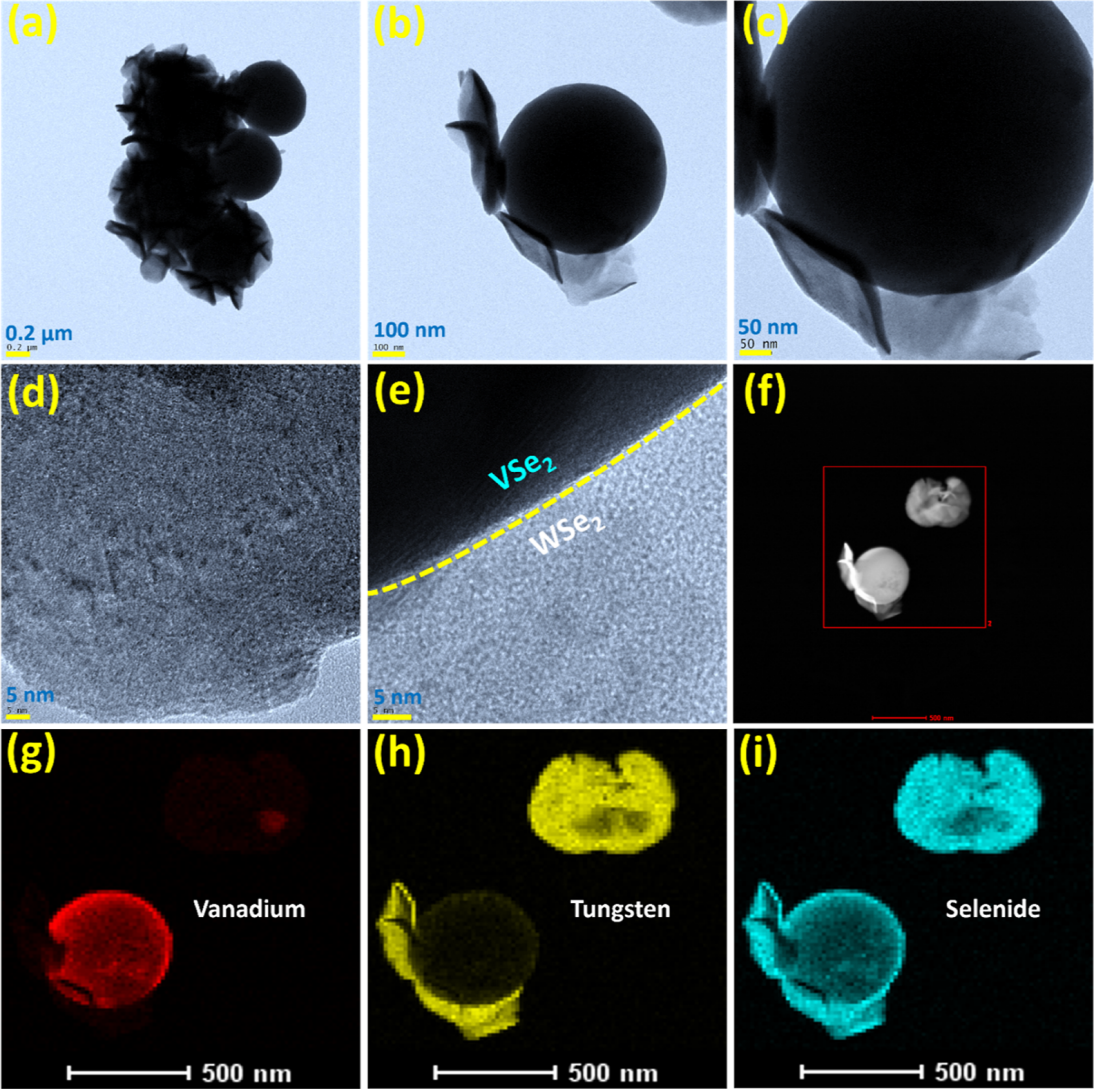
Different magnified TEM images of (a–e) VSe_2_/WSe_2_ hybrid and (f) HADDF-STEM image, along with corresponding elemental distribution patterns of (g) V, (h) W, and (i) Se in the VSe_2_/WSe_2_ hybrid.

After structural confirmation, the morphological features of the 3D/2D VSe_2_/WSe_2_ hybrid were confirmed by both FE-SEM and TEM analyses. Figure 2a–c shows the different magnified FE-SEM images of pristine WSe_2_, which appear as bundles of small nanosheets. These very dense WSe_2_ nanosheets with relatively thin and smooth surfaces are interconnected. As expected, the multidimensional architecture of VSe_2_/WSe_2_ consists of 2D nanosheets and 3D microspheres, Figure 2d–f. It can be seen from the figure that a larger number of WSe_2_ nanosheets are randomly arranged on the surface of the VSe_2_ microspheres. Figure 2g,h shows the EDAX spectra of WSe_2_ and the VSe_2_/WSe_2_ hybrid. The spectra show only the presence of W and Se in WSe_2_ and V, W, Se in the VSe_2_/WSe_2_ hybrid without other impurities. Apart from these elements, strong Cu and Pt signals were observed due to the Cu substrate and Pt sputtering. The magnified TEM images in Figure 3a–c also show that the VSe_2_ microspheres are surrounded by many ultrathin and transparent WSe_2_ nanosheets. Moreover, some of the microspheres can be seen between the nanosheets, proving the strong structural integrity between the multidimensional architectures. Moreover, the presence of highly transparent WSe_2_ nanosheets (as seen on the high-resolution image (Figure 3d,e) indicates very low stacking after combination with VSe_2_ and is consistent with the p-XRD results. These unique structural features of WSe_2_ are favorable for exposing more active edge sites and reducing charge transfer resistance, which enables high catalytic activity for conducting electrochemical reactions at the surface. In addition, the close contact between the unique 3D/2D structures provides an abundance of electroactive sites and facilitates electron transport during the electrochemical reaction at the electrode–electrolyte interface. In addition, the high-resolution lattice image in Figure 3d,e shows that the combinations of the multidimensional architecture of 2D WSe_2_ nanosheets covered with 3D VSe_2_ microspheres create a heterointerface (polymorph) at the lattice. Once the structure was confirmed, the elemental distribution was determined using annular high angle dark field imaging (HADDF-STEM) and area scan mapping analysis. The HADDF-STEM image is shown in Figure 3f and confirms the presence of a dark-field resolved 3D/2D VSe_2_/WSe_2_ structure. The surface mapping images of all elements present in VSe_2_/WSe_2_ are shown in Figure 3g–i. Elements such as W, V, and Se are clearly recognized in the corresponding mapping areas. The mapping results also confirm that all elements are evenly distributed in the hybrid system. In summary, the morphological studies show the successful formation of a 3D/2D VSe_2_/WSe_2_ hybrid structure by a simple solvothermal approach.

### 3.2 Electrochemical Analyses of VSe_2_/WSe_2_ Hybrids

Electrochemical studies were carried out on the VSe_2_/WSe_2_ hybrids using an SPE (VSe_2_/WSe_2_/SPE) to evaluate their potential as an electrochemical sensor for 5-NQ and to gain insight into the mechanism of the 5-NQ reduction process. For comparison, similar data were recorded for the single components, VSe_2_/SPE and WSe_2_/SPE.

#### 3.2.1 A Comparison of VSe_2_/SPE, WSe_2_/SPE and VSe_2_/WSe_2_/SPE

**Figure 4:**
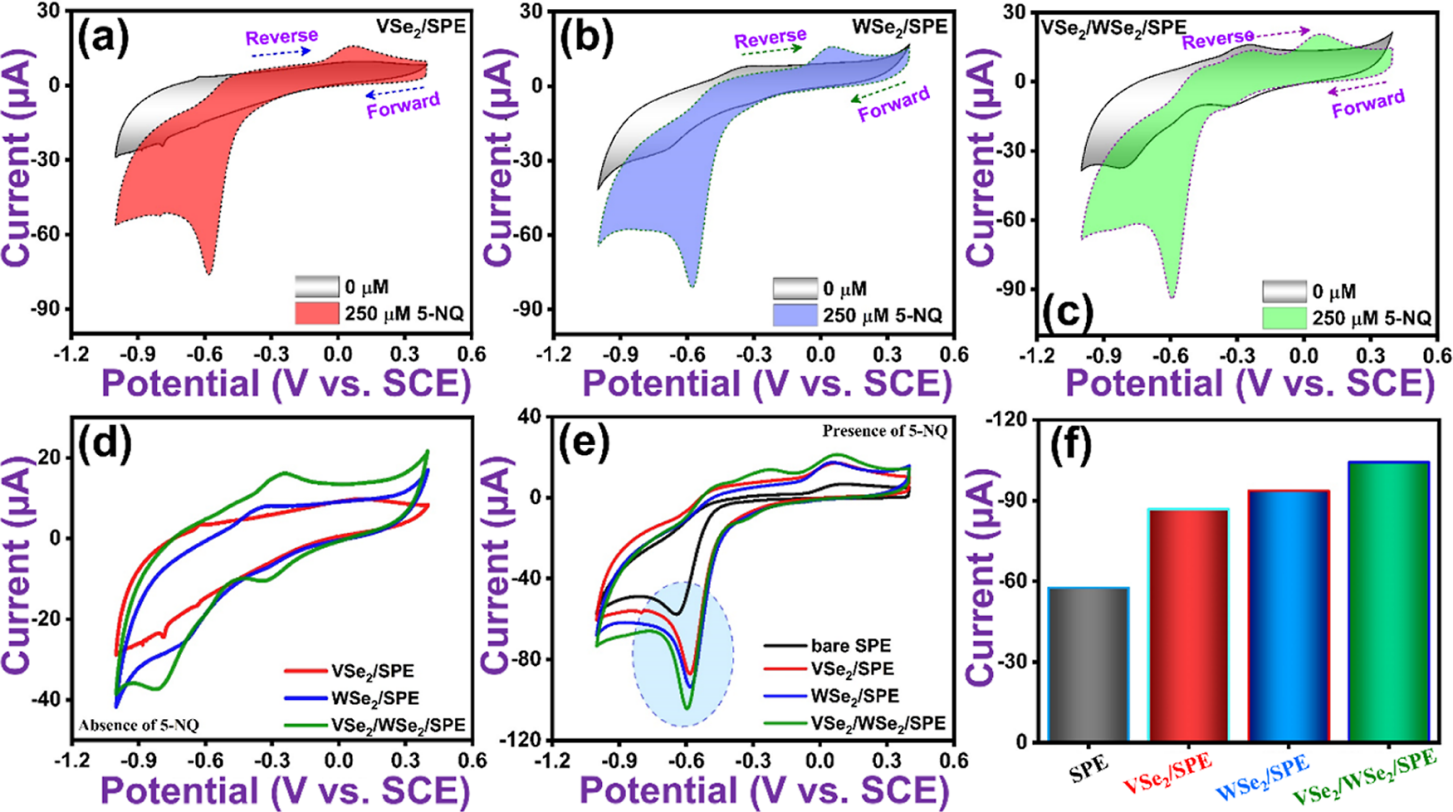
Separate CV curves recorded for (a-c) in the absence and presence of 250 μM 5-NQ at VSe_2_/SPE, WSe_2_/SPE, and VSe_2_/WSe_2_/SPE. Combined CV curves for (d) the absence and (e) the presence of 300 μM 5-NQ at SPE, VSe_2_/SPE, WSe_2_/SPE, and VSe_2_/WSe_2_/SPE. (f) Bar graph comparing SPE, VSe_2_/SPE, WSe_2_/SPE, and VSe_2_/WSe_2_/SPE in the detection of 5-NQ. Conditions: 0.05 M PB (pH 7.0); scan rate 50 mV s^–1^.

**Scheme 2:**
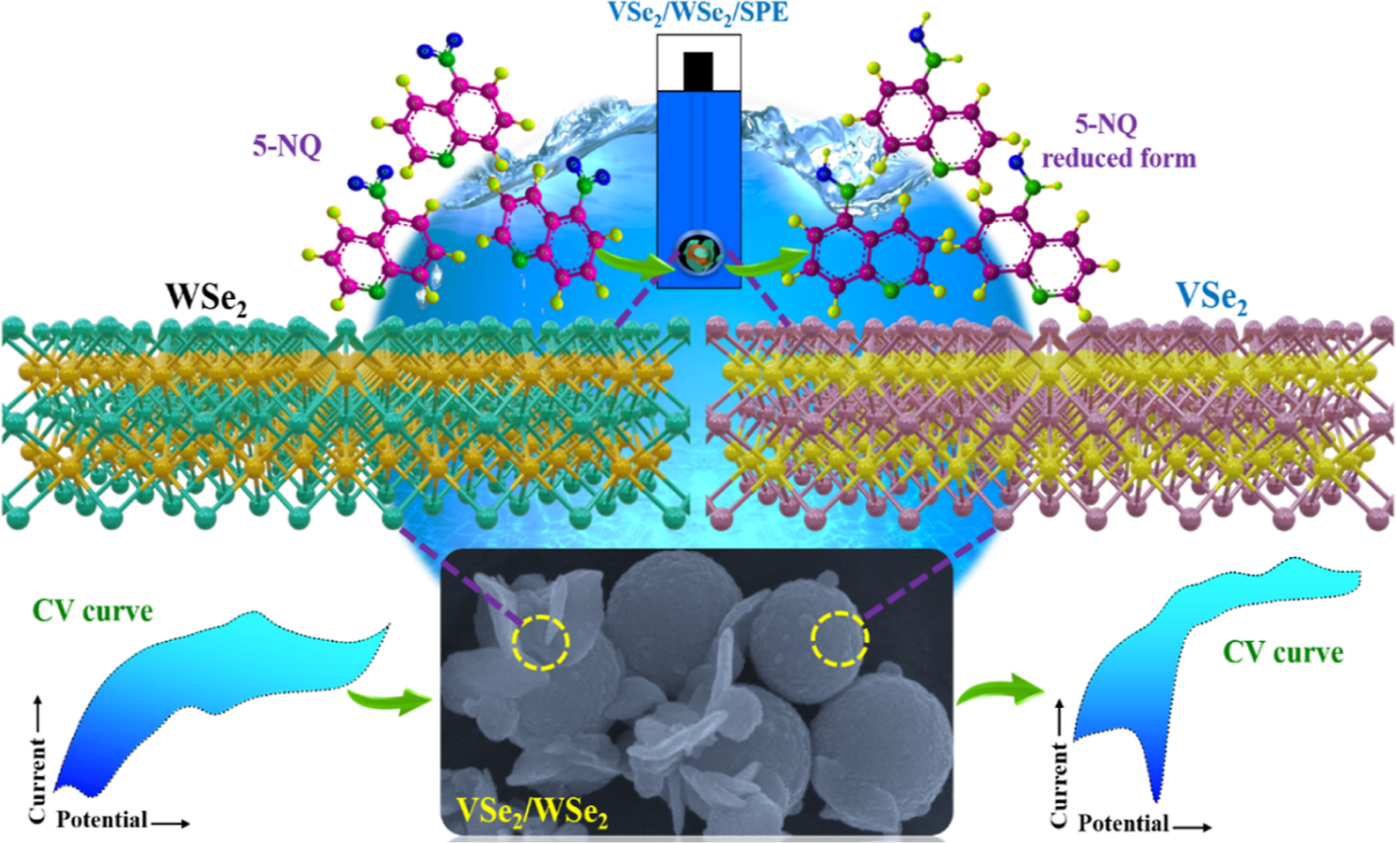
Electrochemical Reduction Mechanism of 5-NQ on the VSe_2_/WSe_2_ Hybrid Modified SPE

**Figure 5:**
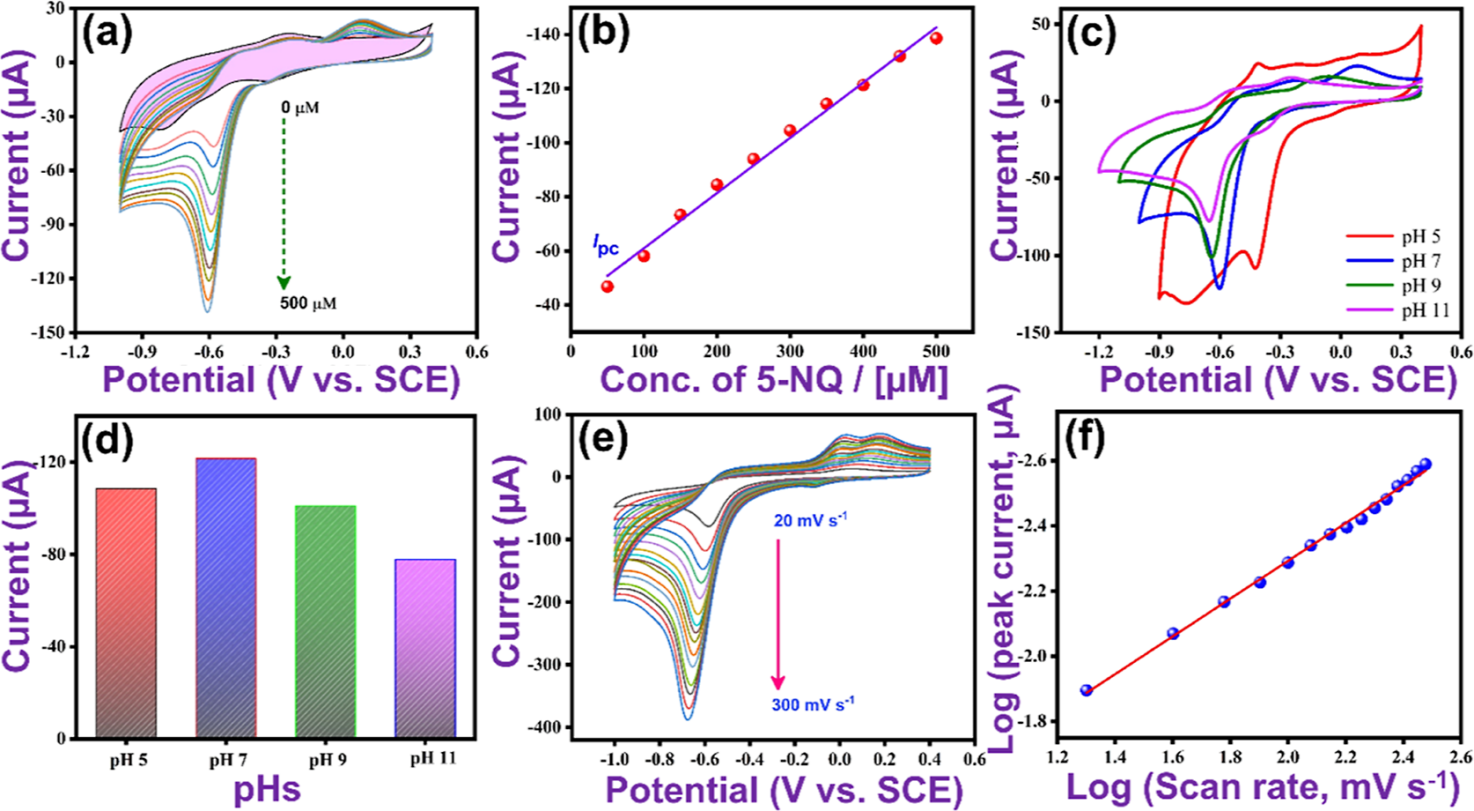
(a) CVs for VSe_2_/WSe_2_/SPE at different concentrations of 5-NQ (0–500 μM), (b) the corresponding linear plot, (c) CVs at different pH values (5–11; 0.05 M PB), (d) corresponding bar graph for peak current as a function of pH for 5-NQ (300 μM). (e) CV curves for VSe_2_/WSe_2_/SPE at different scan rates (20–300 mV s^–1^), and (f) logarithm of the peak current as a function of the logarithm of the scan rate.

The performance of the VSe_2_ (Figure 4a), WSe_2_ (Figure 4b) and VSe_2_/WSe_2_ (Figure 4c) modified electrodes and bare SPE (Figure 4e) in the electrochemical reduction of 5-NQ is shown in Figure 4a–e, where the potential is cycled from 0.4 to −1.0 V at a scan rate of 50 mV s^–1^, in the presence of 250 and 300 μM 5-NQ (0.05 M PB, pH 7). The voltammograms are characterized by a sharp reduction wave in the vicinity of −0.6 V, which can be attributed to the irreversible transfer of four electrons and four protons corresponding to the conversion of the nitro group, NO_2_, to the hydroxylamine group, NHOH, as illustrated in eq 1, and the schematic electrochemical reduction mechanism of 5-NQ on the SPE modified with VSe_2_/WSe_2_ hybrids is shown in Scheme 2. Additional peaks are evident, with oxidation waves at approximately −0.15 and 0.30 V and a small reduction wave at about −0.30 V. To determine the nature of these additional redox waves, CVs were recorded in the absence of the 5-NQ and these are presented in Figure 4d. Here it is clear that the VSe_2_/WSe_2_/SPE exhibits a redox reaction, with an oxidation wave evident at −0.24 V, while the corresponding reduction wave occurs at about −0.34. The small peak-to-peak separation indicates that this redox reaction is confined to the surface, and possibly is related to a reversible change in the oxidation state of tungsten, between W(IV) and W(VI).^29^ This event is not seen with VSe_2_, however, when the VSe_2_ is combined with WSe_2_ to form a hybrid, the redox waves become more pronounced indicating that the WSe_2_/VSe_2_ hybrid facilitates electron transfer. Another broad reduction wave is evident at about −0.81 V for the WSe_2_/VSe_2_ hybrid. This is not evident with VSe_2_, but is seen with WSe_2_ and becomes more pronounced with the WSe_2_/VSe_2_ hybrid. There is no evidence of a corresponding oxidation wave, and consequently, it is unlikely to be connected to the reduction of W(IV) to a lower oxidation state, such as W(III) or W(II). Instead, it may be related to the reduction of hydrogen ions and the evolution of hydrogen gas molecules. Indeed, on increasing the acidity of the solution, a much larger reduction wave was evident at these low potentials (see Figure 5c).

On comparing Figure 4d,e, the oxidation wave at 0.07 V appears to be related to the oxidation of the hydroxylamine, Ar-NHOH, as described in eq 2 following the reduction reaction, eq 1.^30^ A comparison of the four electrodes in the determination of 5-NQ is shown in Figure 4f, where it is clearly evident that the VSe_2_/WSe_2_/SPE has the greatest potential in the development of an electrochemical sensor for 5-NQ, reaching the highest peak current of −104.7 μA (*I*_pc_). Accordingly, all the remaining studies were carried out with this VSe_2_/WSe_2_/SPE.
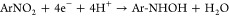
1
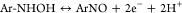
2

The influence of the concentration of 5-NQ on both the reduction peak at −0.6 V, corresponding to eq 1, and the oxidation peak at 0.07 V, eq 2, is shown in Figure 5a. As the concentration increases, both the reduction and oxidation waves increase, with the greatest increase in the reduction peak current. The corresponding linear plot, showing the relationship between the concentration of 5-NQ and the reduction peak current, is shown in Figure 5b. The linear regression equation, *I*_pc_ (μA) = −0.20 *c* (μM) – 40.52, (*R*^2^ = 0.992), was obtained, clearly highlighting that the VSe_2_/WSe_2_/SPE is efficient in the reduction of 5-NQ. To validate this effect, we conducted electrochemical sensing of 5-NQ using varying molar ratios of V in the VSe_2_/WSe_2_/SPE under identical conditions. The resulting CV curves are presented in Figure S2. Analysis of these curves clearly indicates that the lower molar ratio (1:1) of V in VSe_2_/WSe_2_/SPE exhibits a heightened current response for 5-NQ detection compared to the higher ratio (1.5:1). As a result, we opted for the 1:1 ratio of VSe_2_/WSe_2_/SPE for the electrochemical detection of 5-NQ.

#### 3.2.2 Influence of pH and Scan Rate

Since the reduction of 5-NQ involves the transfer of protons, the pH of the solution will have an influence on its electrochemical reduction and detection. The pH was varied from 5.0 to 11.0, and representative CVs, recorded at pH values of 5.0, 7.0, 9.0, and 11.0 are shown in Figure 5c. The voltammograms recorded at pH values from 7.0 to 11.0 have a similar shape, with the main reduction peak arising from the conversion of the nitro to the hydroxylamine groups. However, the voltammogram recorded at pH 5.0 differs, with higher background currents and a large broad reduction wave occurring at potentials in the vicinity of −0.80 V, in addition to the reduction of 5-NQ being shifted to a potential of −0.42 V in this acidic solution. The large cathodic currents evident at the lower potentials may be associated with the hydrogen ion (H^+^) reduction reaction and the evolution of gaseous hydrogen (H_2_(g)) molecules. Accordingly, lower pH values were not employed as the peak current arising from the reduction of 5-NQ in these acidic solutions was too difficult to resolve with the competing hydrogen evolution reaction. The influence of pH on the peak current is summarized in the form of a bar graph (see Figure 5d). The optimum pH is clearly at a pH of 7.0, which corresponds to a neutral solution. Figure S3 also shows that the peak potential depends on the pH, with the peak potential increasing rapidly from a pH of 5.0 to 7.0 and only slightly from 7.0 to 11.0.

In Figure 5e, the influence of scan rate on the reduction of 5-NQ is shown. On increasing the scan rate from 20 to 300 mV s^–1^ in the presence of 5-NQ containing 0.05 M PB (pH 7), the peak current increases, while the peak potential shifts to more negative potentials. This is consistent with a somewhat slow electron transfer reaction. These scan rate studies were further analyzed to determine if the reduction of 5-NQ is under diffusion or adsorption control. In this context, the peak current associated with the reduction of 5-NQ was plotted as a function of both the scan rate and the square root of the scan rate. Linear plots, Figure S4a,b, were obtained in both cases, with linear regression equations of *I* (μA) = −1.04 ν (mV s^–1^) – 80.15 (*R*^2^ = 0.991) and *I* (μA) = −23.64 ν^1/2^ (mV s^–1^)^1/2^ – 38.6 (*R*^2^ = 0.989), corresponding to eq 3 (adsorption) and (eq 4) (Randles–Ševcík equation).^31^ In these relationships, *F*, *R*, and *T* have their usual meanings, α is the charge-transfer coefficient, *A* represents the surface area, τ* corresponds to the surface-bound redox couple, *D* is the diffusion coefficient of the analyte, and *c* is the bulk solution concentration of the analyte (these equations are written for a one-electron transfer reaction). The nonzero intercepts indicate deviations from these ideal relationships. To differentiate between adsorption and diffusion control, the logarithmic of the peak current was plotted as a function of the logarithm of the scan rate. This plot is shown in Figure 5f, with a linear regression equation of log *I* (*I* (μA)) = 0.579 log *v* (*v* (mV s^–1^)) + 1.13, with an *R*^2^ value of 0.996, indicating excellent linearity. This slope of 0.579, being close to a value of 0.50, suggests a mainly diffusional controlled reduction of 5-NQ.
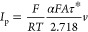
3
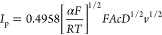
4

#### 3.2.3 Analytical Performance of the VSe_2_/WSe_2_/SPE

**Figure 6:**
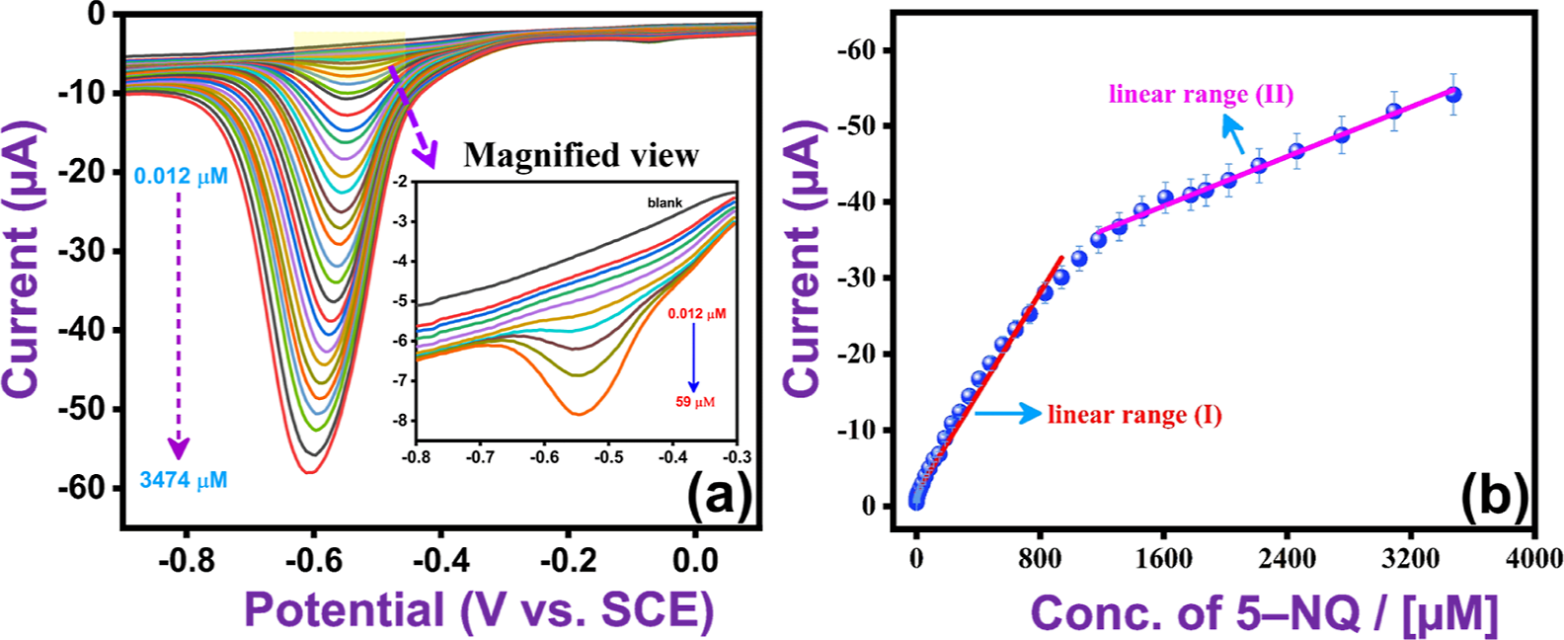
(a) DPVs shown as a function of the 5-NQ concentration from 0.012 to 3474 μM, and (b) calibration curves over an extended concentration range.

The analytical performance of VSe_2_/WSe_2_/SPE was evaluated using DPV; a typical plot is shown in Figure 6a. In these studies, the concentration varied from a relatively low concentration of 12 nM to a high concentration of 3.474 mM. There is a clear increase in the peak current as the concentration is increased and when the peak current was plotted as a function of concentration, two linear regions were obtained as shown in Figure 6b. The linear regression equations for the lower (0.012–1053 μM) and higher (1183–3474 μM) concentration ranges are *I*_p_ (μA) = 0.033 *c* (μM) + 1.71 (*R*^2^ = 0.988), and *I*_p_ (μA) = 0.008 *c* (μM) + 26.43 (*R*^2^ = 0.992), respectively. Using the lower linear concentration region, which extends from 0.012 to 1053 μM with a sensitivity of 0.47 μA/μM/cm^2^, the limit of detection (LOD) was computed as 2 nM, where LOD = 3σ/*S* (σ stands for the standard deviation of the blank solution and *S* for the slope of the calibration plot from the lower linear response range). These analytical parameters compare very favorably to previously reported sensors for the detection of 5-NQ, as shown in Table S1. Clearly, the VSe_2_/WSe_2_/SPE has a more extended linear range and a lower LOD than these recently reported sensors.

**Figure 7:**
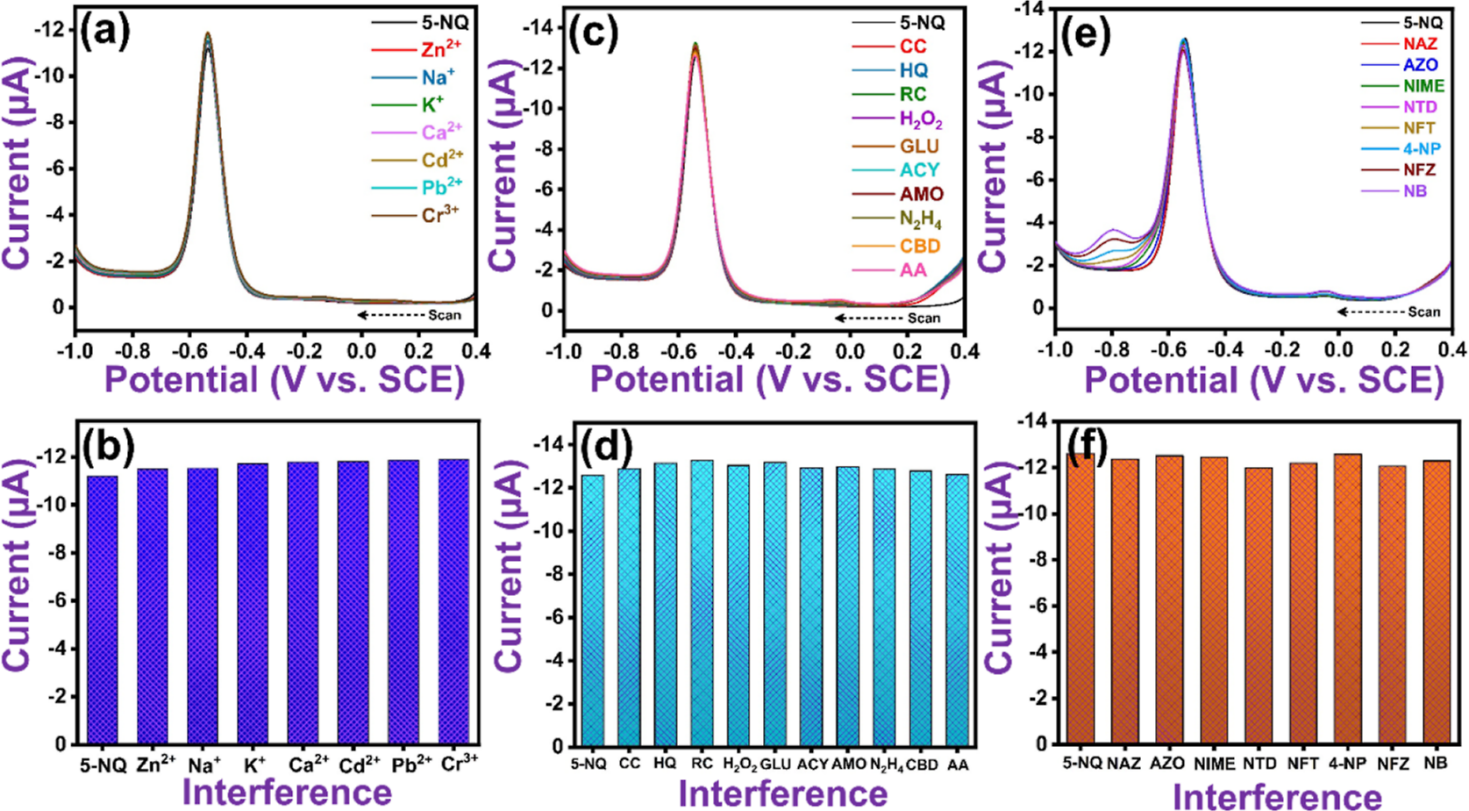
(a,c,e) DPVs recorded for 5-NQ and the addition of various interferents (cations, non-nitro- and nitro group-containing drugs, pollutants, and biomolecules), (b,d,f) the corresponding current response for the selected interferents, relative to the 5-NQ in the absence of the interferents.

Selectivity studies play a crucial role in sensor development, as many structurally similar compounds or common ions can significantly interfere with the accurate determination of 5-NQ. The selectivity of VSe_2_/WSe_2_/SPE was evaluated by adding various interfering substances, such as common ions, biomolecules, and nitro group/non-nitro group containing drugs/pollutants to the solution containing 150 μM 5-NQ (0.05 M PB, pH 7). The interferents were also set at a concentration of 150 μM. The DPV curves obtained and the current response to these interferents in terms of bar graphs are shown in Figure 7a–f. In Figure 7c,d, DPV responses are shown for 5-NQ in the absence and presence of an equal concentration of interfering compounds (biomolecules/pollutant/non-nitro group containing drugs), including catechol (CC), hydroquinone (HQ), resorcinol (RC), hydrogen peroxide (H_2_O_2_), glucose (GLU), acyclovir (ACY), amoxicillin (AMO), hydrazine (N_2_H_4_), carbendazim (CBD), and ascorbic acid. The study revealed no interference from these compounds on the developed 5-NQ sensor, with the maximum change in peak current remaining below 5%. Moreover, selectivity was assessed with an equal concentration (150 μM) of various common ions, such as Zn^2+^, Na^+^, K^+^, Ca^2+^, Cd^2+^, Pb^2+^, and Cr^3+^ (Figure 7a,b). As shown in Figure 7a,b, good selectivity was achieved. There is a small increase in the peak current upon the addition of the cations, with a somewhat higher current observed with Cr^3+^. This may be due to an increase in the conductivity of the 0.05 M PB solution. However, the change in peak current remains less than 5.6% for all these inorganic ions. The DPVs presented in Figure 7e,f) for 150 μM 5-NQ in the presence of an equal concentration of various nitro-containing analytes, such as nitazoxanide (NAZ), azithromycin (AZO), nimesulide (NIME), nitrendipine (NTD), nitrofurantoin (NFT), 4-nitrophenol (4-NP), nitrofurazone (NFZ), and nitrobenzene (NB), clearly show that the peak current associated with 5-NQ is not affected by any of these additives, with the maximum change in peak current remaining below 3%. Interestingly, several of these interferents are reduced at the VSe_2_/WSe_2_/SPE, but the potential difference between the peak potential of 5-NQ and the interferents, such as NFT, 4-NP, NFZ, and NB (more than −0.7 V), is sufficiently large to allow simultaneous detection of 5-NQ and these analytes. This makes the VSe_2_/WSe_2_/SPE sensor even more versatile. The stability of the VSe_2_/WSe_2_/SPE sensor is illustrated in Figure S5, where the sensor was used over 33 repeated measurements at a relatively high concentration (250 μM) of 5-NQ using the CV technique. These high concentrations were chosen because the surface of a sensor can easily become poisoned at such high concentrations. Very good stability was achieved, with the current changing by only 6% over 33 repeated measurements.

## 4 Conclusions

In summary, a 3D/2D VSe_2_/WSe_2_ hybrid was successfully prepared by simple solvothermal synthesis. This hybrid has superior electrochemical properties compared to the individual components. The fabricated SPE modified with the VSe_2_/WSe_2_ hybrid shows excellent electrochemical activity for the detection of 5-NQ with a low detection limit and good sensitivity as well as good selectivity and repeatability. It appears that the weak van der Waals forces between the WSe_2_ layers, which lead to stacking and a reduction in the number of active sites, can indeed be overcome by forming a hybrid with VSe_2_. This hybrid consists of 2D WSe_2_ nanosheets dispersed over 3D VSe_2_ microspheres that serve to isolate the WSe_2_ nanosheets. This results in the exposure of a much larger number of WSe_2_ edges and active sites. This hybrid not only exhibits efficient electron transfer but shows very good long-term stability. All these promising results make the VSe_2_/WSe_2_-based electrodes an ideal platform for electrochemical applications.

## Supporting Information Available

Reference powder XRD patterns for VSe_2_/WSe_2_ adapted from the ICSD database, and XRD diffractograms of VSe_2_/WSe_2_ hybrids with 1:1 and 1.5:1 of V, the linear plot for the cathodic peak potential versus different pH values, the linear plots for the reduction peak current of 5-NQ versus both the scan rate and the square root of the scan rate, comparison of the performance of the proposed electrode with previously reported electrochemical 5-NQ sensors, CV curves for 5-NQ detection using 1:1 and 1.5:1 ratios of V in VSe_2_/WSe_2_/SPE, bar graph for the stability over repeated cycles at a 5-NQ concentration of 250 μM at VSe_2_/WSe_2_/SPE (PDF)
