# Supplementary material for: Synergistic Effect of 3D/2D Vanadium Diselenide/Tungsten Diselenide Hybrid Materials: Electrochemical Detection of 5-Nitroquinoline a Hazard to the Aquatic Environment
Source: ACS Appl Mater Interfaces. 2024 Jun 17;16(26):33325–35. doi: 10.1021/acsami.4c02412 (PMC11231969; doi:10.1021/acsami.4c02412)
Supplement: Supplementary file 1 — am4c02412_si_001.pdf [file am4c02412_si_001.pdf]

Supporting Information

Synergistic Effect of 3D/2D Vanadium Diselenide/Tungsten Diselenide Hybrid Materials: Electrochemical Detection of 5-Nitroquinoline Hazardous to the Aquatic Environment

Ramaraj Sukanya[a,1], Prajakta R. Chavan[b,1], Raj Karthik[c,b*], Mahmudul Hasan[b], Jae-Jin Shim[b,*], Carmel B. Breslin[a,*]

[a] Department of Chemistry, Maynooth University, Maynooth, Co. Kildare, W23F2H6, Ireland

[b]School of Chemical Engineering, Yeungnam University, Gyeongsan, Gyeongbuk 38541, The Republic of Korea.

[c]Centre of Molecular Medicine and Diagnostics (COMManD), Department of Biochemistry, Saveetha Dental College and Hospitals, Saveetha Institute of Medical and Technical Sciences (SIMATS), Saveetha University, Chennai 600 077, India

Corresponding Authors:

Prof. Jae-Jin Shim (J.J. Shim)

E-mail: jjshim@yu.ac.kr

Dr. Raj Karthik (R. Karthik)

E-mail: jackeykarthik@gmail.com

Prof. Carmel B Breslin (C.B. Breslin)

E-mail: carmel.Breslin@mu.ie

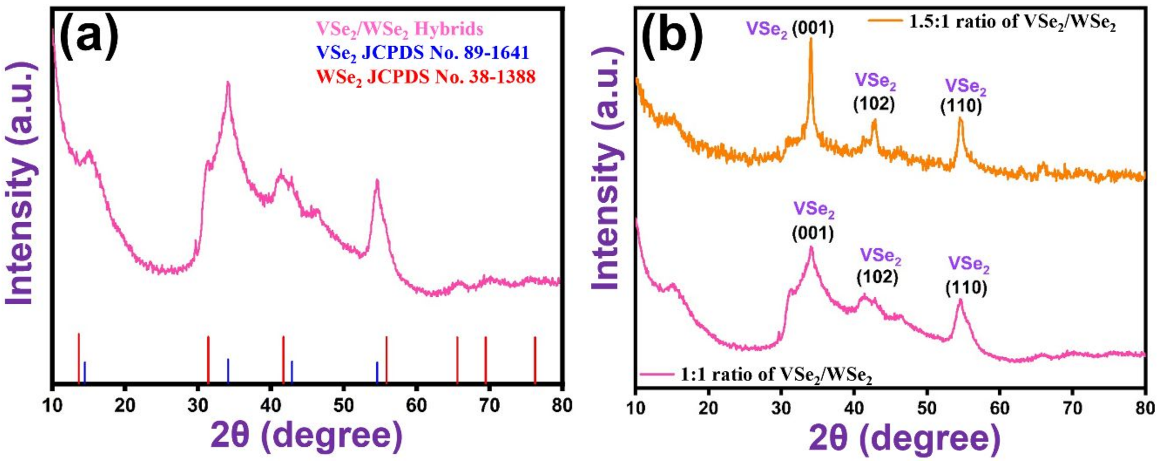

Figure S1. (a) Reference powder XRD patterns for VSe_2/WSe_2 adapted from the ICSD database, and (b) XRD diffractograms of VSe_2/WSe_2 hybrids with 1:1 and 1.5:1 of V.

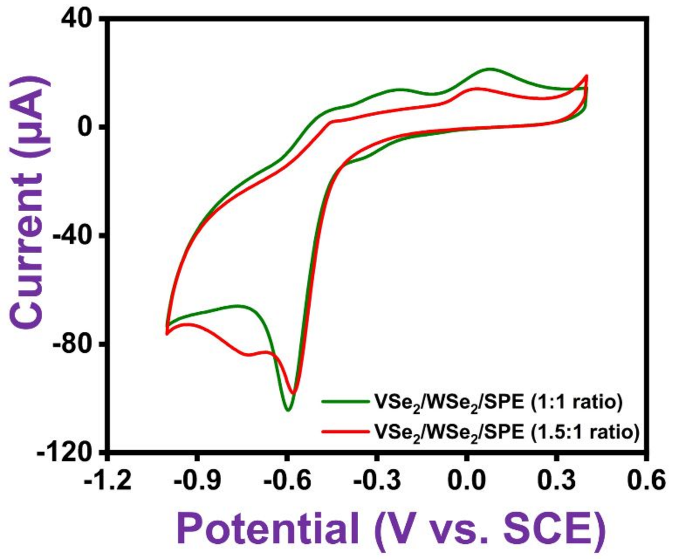

Figure S2. CV curves for 5-NQ detection using 1:1 and 1.5:1 ratios of V in VSe_2/WSe_2/SPE.

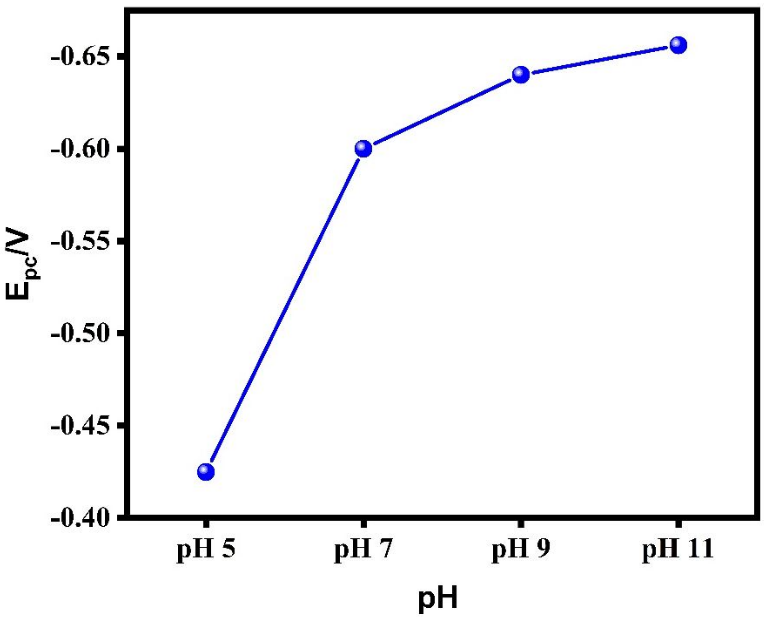

Figure S3. The linear plot for the cathodic peak potential vs. different pH values

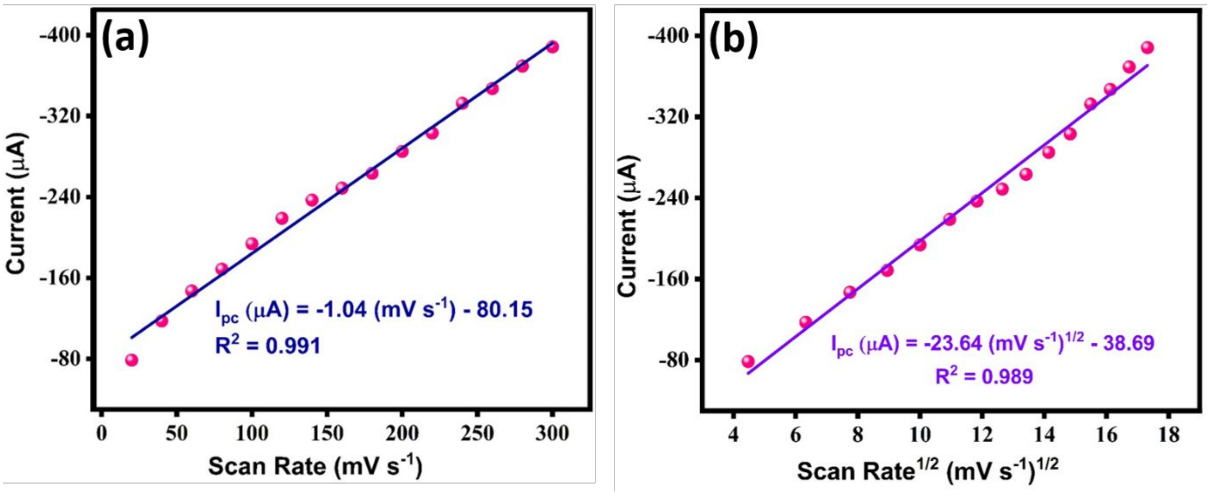

Figure S4. The linear plots for the reduction peak current of 5-NQ vs. function of both the (a) scan rate and (b) the square root of the scan rate.

Table S1. Comparison of the performance of the proposed electrode with previously reported electrochemical 5-NQ sensors.

Working Electrode	Method	Linear range	LOD	Ref.
HmDmE	DPV	20-1000 mol/L	Not given	[19]
m-AgSAE	DPV<br>FIA	0.2-1000 mol/L<br>-	Not given<br>3 μM/L	[18]
BDD	DPV	0.5-75 μM dm^{-3}	0.5 μM	[32]*
CFE	DPV	0.4-100 mol dm^{-3}	Not given	[21]
2D-CoSe/Ni_3B/SPCE	DPV	0.2 - 378 μM	0.042 μM	[20]
**VSe_2/WSe_2/SPE**	**DPV**	**0.012-1053 μM,<br>1183-3474 μM**	**0.002 μM**	**This Work**

* Yosypchuk, O.; Karasek, J.; Barek, V.V.; Peckov, K. The use of silver solid amalgam electrodes for voltammetric and amperometric determination of nitrated polyaromatic compounds used as markers of incomplete combustion. *The Scientific World J*, **2012**, 231986.

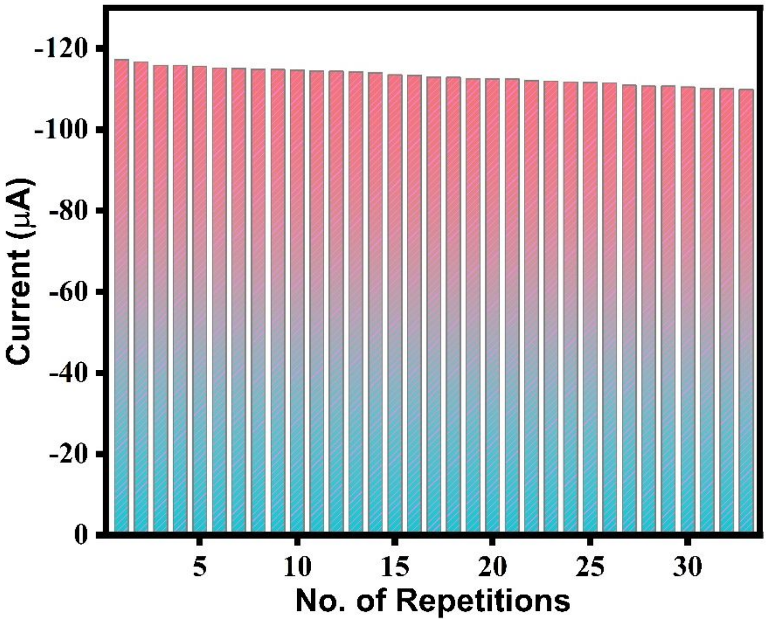

Figure S5. Bar graph for the stability over repeated cycles at a 5-NQ concentration of 250 μM at VSe_2/WSe_2/SPE.
